# Participatory Autism Research: How Consultation Benefits Everyone

**DOI:** 10.3389/fpsyg.2021.713982

**Published:** 2021-08-24

**Authors:** Connor Tom Keating

**Affiliations:** School of Psychology, University of Birmingham, Birmingham, United Kingdom

**Keywords:** autism, community, involvement, methods, participatory research

## Introduction

In the past decade, there has been increasing concern about the disconnect between researchers and the autism community (autistic people and their family members) (Pellicano and Stears, 2011; Milton and Bracher, 2013; Milton, 2014; Chown et al., 2017; Woods and Waltz, 2019). This disconnect may be due to a number of factors, including a lack of involvement of the autism community in research (Gowen et al., 2019), rare (or non-existent) dissemination of findings to the community, and use of demeaning language about autistic people in scientific works (Gowen et al., 2019). This, alongside a history of controversial claims from scientists (from “refrigerator mothers” to claims vaccines cause autism) has contributed to growing distrust of autism researchers by autistic self-advocates (Dawson, 2004; Bagatell, 2010).

Fortunately, there is a solution—participatory research. Participatory research involves incorporating the views of the autism community about what research gets done, how it is done and how it is implemented (Cornwall and Jewkes, 1995). Specific manifestations of participatory research include “*leadership* by autistic researchers, *partnership* with autistic people or allies in research, *engagement* with the community (e.g., via social media) and *consultation* with relevant individuals or community organizations” (Fletcher-Watson et al., 2019). In addition, an important component to participatory working is making research accessible to *all* members of the autism community—for instance by adapting the research environment (see Pellicano et al., 2017), methodology and dissemination routes to permit the widest engagement and inclusion of under-represented groups in research (e.g., non-speaking autistic individuals and people with co-occurring learning disabilities).

Another key principle of participatory research is the acknowledgment, and undermining, of the power imbalance between researcher and participant (Nelson and Wright, 1995). One way to conceptualize this power imbalance is using the *ladder of participation* (Arnstein, 1969), which outlines that power varies across different types of participation: from no power (e.g., recipient of therapy), through tokenism (e.g., informing after instead of consultation in advance) to devolved power (e.g., partnership and citizen control), where planning and decision-making are shared. Researchers should aim to level the traditional power imbalance by adopting participatory practices and, in their reporting of community involvement, highlight the power dynamics involved (Pickard et al., 2021).

### Why Is Participatory Research Important?

There are a multitude of benefits of participatory research. Community input can (a) improve the quality of research methods and place findings within a real-world context, thus facilitating the translation of findings into practice (Grinker et al., 2012; Parsons and Cobb, 2013; Carrington et al., 2016; Fletcher-Watson et al., 2019), (b) ensure that research yields relevant and meaningful benefits for the autism community (Long et al., 2017), and (c) enhance involvement, collaboration and trust between researchers and autistic people and their allies (Gowen et al., 2019). Despite a multitude of benefits, unfortunately, there is evidence to suggest that participatory research is not yet the standard, but rather the exception.

### How Common Is Participatory Research?

At present, it is thought that a large proportion of autism research involves no community participation or only tokenistic participation of the autism community (Nicolaidis et al., 2011; Fletcher-Watson et al., 2019). Indeed, the UK report “*A Future Made Together”* (Pellicano et al., 2013), elucidated that opinions on the prevalence of participatory research were contrasting—whilst autism researchers perceived themselves to be engaged with the autism community (e.g., dissemination and consultation), autistic people and their families did not share this view (Pellicano et al., 2014). This report also highlighted that research funding and output in the UK is not in line with the priorities of autistic people, their families and practitioners, with two-thirds responding that they were either *dissatisfied* or *very dissatisfied* with current spending/output.

Unfortunately, in the rare circumstances where there is autism community involvement, at present, this is rarely more than tokenistic (Fletcher-Watson et al., 2019; Michael, 2021). In other words, some researchers will adopt a participatory approach in order to “tick a box” (i.e., to meet a funder, journal of ethics board requirement) rather than to provide the opportunity for the autism community to *actually* influence outcomes. At best, these tokenistic approaches may fail to deliver meaningful results for the community, and at worst, they are insulting and damage the relationship between autistic people and academics, thus leading to non-participation in research (Fletcher-Watson et al., 2019). This is exemplified in the experiences of Cos Michael, who has reported that they have sometimes felt like they were the “token autistic” and have subsequently given up on university-based autism research (Michael, 2021). In order to avoid tokenism, researchers should work with community members who have expertise and experiences relevant to the topic of research; actively listen and learn from this expertise and make changes based on feedback; and recognize the power imbalance in most research scenarios (Fletcher-Watson et al., 2019).

### Why Is Effective Participatory Research Not Happening?

There are multiple factors that can complicate attempts to adopt collaborative research practices (Pickard et al., 2021; Redman et al., 2021). One key reason is that the infrastructure of scientific research is not conducive to participatory working in a number of ways (Fletcher-Watson et al., 2019; Pickard et al., 2021). Firstly, there are significant time and funding constraints within academic environments that may prohibit a participatory working style (Fletcher-Watson et al., 2019; Pickard et al., 2021). Secondly, participatory approaches are not incentivized, for instance in terms of career progression, within the current academic structure (Pickard et al., 2021). Finally, some early career researchers feel that there is an absence of support for participatory working from more senior academics (Pickard et al., 2021). Therefore, systemic change is necessary to ensure that participatory practices can be accommodated within current research frameworks.

Another reason that researchers may not adopt a participatory approach is due to challenges relating to objectivity and methodology. Some researchers have raised concerns that objectivity could be compromised through engagement with autistic partners (see Pellicano et al., 2014). However, as Fletcher-Watson et al. (2019) highlight, “serious biases—for example, towards maintenance of the status quo—can occur when research takes place without community influence.” Alternatively, some researchers may be concerned that autistic people will say something they disagree with or ask them to do something that is not easy to implement. However, as other researchers have highlighted “the irony of this should be obvious: researchers have been asking autistic people to put up with both of these for decades” (Fletcher-Watson et al., 2019).

In addition, researchers may not engage in participatory practices because they believe effective participatory research requires them to have strong relationships with the community, and forging these relationships takes time (Pickard et al., 2021). However, as one academic explained, investing this time is hugely valuable as it can foster “an extremely powerful interpersonal connection or relationship with people for whom participation had never been very meaningful” (Pickard et al., 2021). Ironically, participatory research can enhance rapport and trust between researchers and the autism community (Gowen et al., 2019), and therefore comprises a strategy, in itself, to improve the relationships that are seen as necessary for effective participatory working. Consequently, researchers should adopt a participatory approach imminently to facilitate the formation of these constructive alliances, thereby improving the efficacy of their collaborations with the autism community in the long-term.

### A Brighter Future

Fortunately, more recently there has been increasing recognition that it is time for change, with autistic advocates, academics and activists insisting that participatory research is the way forward (e.g., Pellicano and Stears, 2011; Milton and Bracher, 2013; Fletcher-Watson et al., 2019; Gowen et al., 2019). Indeed, there are some great examples emerging of collaborations that have involved the autism community in priority-setting and research (e.g., Nicolaidis et al., 2011, 2013; Bertilsdotter Rosqvist, 2019; Crane et al., 2019; Fletcher-Watson et al., 2019; Vincent, 2019; Young et al., 2019; Pavlopoulou, 2020; Pellicano et al., 2020).

One gold-standard example of how researchers, autistic people and their allies can effectively collaborate is the “*Shaping Autism Research”* seminar series (Fletcher-Watson et al., 2019). During this seminar series, members of the autism community played prominent roles in every event, including as co-applicants for funding, co-convenors, speakers, panelist and discussion group leaders. Crucially, the organizers also ensured that each seminar was as accessible to autistic individuals as possible by creating a suitable sensory environment and providing a quiet space. The authors reduced power inequalities between delegates by including clear terms of reference for participation, so that all contributors had a shared expectation of what the sessions would involve. In all materials, language was selected that characterized autism in neutral terms (e.g., not a disease or misfortune), thus making respect overt and creating a space where all people were equal. From this seminar series, Pellicano et al. (2017) have produced a starter pack for participatory autism research, providing principles for how academics and the autism community can work together to shape research. Since then, other research teams have built on these principles, providing detailed guidelines for researchers to consider in order to increase involvement, collaboration and trust between researchers and the autism community (Gowen et al., 2019). As such, the “*Shaping Autism Research”* seminar series has laid the foundation for more effective participatory research, in which relevant communities and stakeholders can work collaboratively to create a better future for autistic people, together.

Another good practice example is the work of Pavlopoulou (2020), which adopted a participatory approach throughout the entirety of the research process to investigate facilitators of sleep for autistic adolescents. At the onset of the study, a consultation group provided input on study objectives, research design, procedures and tools, ideas for public engagement, and other areas relating to the specifics of the project. This work involved participant-driven data collection, in which participants were asked to take 10–15 photographs and keep notes or drawings for one week of various environments, activities and objects that were related to their sleep (e.g., the place they sleep and its surroundings, activities/objects/people that may help them to fall or stay asleep, etc.), and participant-driven data coding. Following this, the consultation group created visual aids that were then used for dissemination at a community exhibition alongside panel discussions and workshops involving various members of the autism community (parents, psychologists, autistic people, etc.). By adopting an experience-sensitive participatory approach grounded within the lifeworld framework (see Hemingway et al., 2015 and Pavlopoulou, 2020), the authors acknowledged the autistic participants as active agents in research, recognized their autonomy of thought, perspectives and ideas, and facilitated the translation of findings into practice (see Pavlopoulou, 2020).

Moreover, the work of Cassidy et al. (2020a,b, 2021a,b) constitutes a good example of effective participatory research. First, in this program of work, autistic people identified a need for better tools to assess suicidality in autism (Cassidy et al., 2020a, 2021a). Following this, the authors conducted two studies to adapt the suicidal behavior questionnaire to improve the clarity and relevance of the items to autistic adults. In the first study (Cassidy et al., 2020b), three focus groups identified potential issues with the original version of the questionnaire (that was designed for non-autistic adults) and suggested adaptations. Following this, autistic and non-autistic adults completed the initial adapted version of the questionnaire to explore the equivalence of the tool between groups and identify problematic items. In the second study (Cassidy et al., 2021b), the authors completed cognitive interviews, that had been co-designed with an autism steering group, with nine autistic adults to assess the initial adapted version of the questionnaire. After making the necessary changes, a large sample of autistic adults provided qualitative feedback on each item of the original and refined versions of the tool. Following this, a large sample of autistic and non-autistic adults provided feedback on, and completed, the finalized version of the questionnaire. Lastly, a focus group discussed the findings from the project and potential next steps at an open public engagement event, thus providing the community with an active role in the dissemination of findings. As such, this work comprises a gold-standard example of participatory research in which there was extensive involvement of the community throughout the research process. Without this involvement, the authors would not have been able to capture the unique experiences of suicidality in autistic adults, thus rendering the tool less effective.

### Areas to Focus on

Although there is increasing community involvement in research, autistic individuals with communication differences, such as those who are non-speaking or minimally-speaking (Lebenhagen, 2020) and those with a learning disability (Long and Clarkson, 2017) are less well-represented in this movement (e.g., in Fletcher-Watson et al., 2019; Pavlopoulou, 2020 and Cassidy et al., 2021b, the authors noted that their projects were not fully inclusive of these individuals). Importantly, people with communication differences may require personalized support, unique modes of communication, and well-planned engagement for their voices to be heard (Long and Clarkson, 2017; Long et al., 2017; Lebenhagen, 2020). Without the use of these personalized approaches, communication differences can result in autistic people facing exclusion from processes of consultation and research (Long and Clarkson, 2017) due to communicative normativity (see Lebenhagen, 2020).

The work of Long et al. (2017) exemplifies best practice for conducting research with and for autistic people with communication differences. Specifically, this study aimed to gain the perspective of autistic people with learning disabilities on their experiences of support services (for example regarding support for their health and well-being, support for communication and involvement, the presence of low stress service environments, etc.). Importantly, participants were given the opportunity to communicate in a way that accommodated communicative differences—some moved cut-out photographs or symbols cards around, others wrote or drew onto sheets of paper, and others engaged in purely verbal discussion (see Long et al., 2017; Scott-Barrett et al., 2019 for further guidance on accommodating communicative differences). By accommodating these communication differences, the authors were able to better understand the autistic participants' experiences of their support services, thus allowing their voices to be heard and changes to be implemented accordingly. As such, this work has paved the way for greater participation of autistic individuals with communication differences (for example some individuals with learning disabilities or those who are non-speaking or minimally-speaking) in the research process.

## Participatory Research in the Context of Consultation

One particular strategy that is increasingly being used, more broadly, to promote active involvement of autistic individuals and their allies in the research is *consultation* with the autism community. The idea here is that members of a research team consult a group of individuals from the autism community to discuss their research. Community input can be highly valuable at all stages of research: from initial conception of a study, through data collection, all the way to dissemination of scientific messages. To illustrate this point, we will run through the typical stages of the research process and give some (but not exhaustive) examples of how consultation can be useful both for the autism community and for researchers themselves.

### Generating a Research Question

Input at this early stage of the research process may highlight opportunities to align study objectives with community priorities. To demonstrate the utility of community input at this stage, we will discuss the work of Crane et al. (2019). In this study, the ‘community' comprised a group of young autistic people, between the ages of 16 and 25 years, referred to as the myVoice team (from the UK charity Ambitious about Autism). When asked about their priorities for research, the myVoice team unanimously selected mental health in young autistic people, reflecting the views within the wider autism community (Autistica, 2016). Following this, three members of the myVoice team collaborated with a group of academics, as full and equal partners, during all stages (design, implementation, analysis, interpretation and dissemination) of the research process to address their research question. Crucially, gaining their insight from initial conception of the study ensured that the research was relevant and useful outside of academia (Adams et al., 2018), and would have the largest impact on the lives of those who need it most (Pellicano et al., 2014).

### Designing the Study

Insight from the community when designing a study can be highly useful: from input on experimental design to construction of questionnaires or other participant-facing documents. To demonstrate how input from the community can be invaluable at this stage, I will draw on my own experiences of working with an autism consultancy committee^1^ (Birmingham Psychology Autism Research Team Consultancy Committee; B-PART-CC). We have recently started a project that aims to explore the autism-related language preferences of a diverse set of autistic individuals. We had drafted a questionnaire that asked participants about a broad range of autism-related terminology, for instance asking how they believe is best to refer to someone with an autism diagnosis (e.g., person with autism vs. autistic person etc.). Firstly, the group commented on the clarity and length of each of our questions, thus ensuring the questionnaire was clear and accessible to a range of autistic individuals. In addition, through consultation with the group, we identified some additional terms (e.g., “is neurodivergent”), and an additional category of terms, concerning how we refer to non-autistic people (e.g., “typical” vs. “neurotypical” vs. “non-autistic,” etc.). Multiple members of the group made the point that how we refer to people *without* autism is just as important as how we refer to those *with* autism. This is because the terms we use to speak about non-autistic people intrinsically have connotations about autism and autistic people. Accordingly, we have added this category of terms to our questionnaire. Therefore, input at this stage not only improved the clarity of participant-facing documents, but also elucidated a priority for the community (to establish how we should refer to non-autistic people) and broadened the potential impact of our paper.

### Data Collection

At the point of data collection, the autism community could advise on how to create an enabling environment for autistic individuals. For example, they may suggest that you ask each of your participants if they have any specific needs and/or have a preferred way to communicate (e.g., through spoken or written language or symbols and pictures). In my own experience, they may also provide some more general advice like give plenty of warning of any changes to the setting or situation, or to appreciate that not everyone likes eye contact (see Pellicano et al., 2017). They may also identify ways to adjust your study to make it accessible to groups typically under-represented in research (e.g., non-verbal individuals or those with co-occurring learning disabilities), thus making the research and data applicable (and generalizable) to a more diverse range of autistic individuals. Creating an accessible and enabling environment for participants is a necessity—as researchers, we have a duty of care to protect participants and ensure they are as comfortable as possible.

### Dissemination of Findings

During this final stage, community input can facilitate the creation of scientific messages that are maximally accessible to members of the autism community. For example, the community could provide feedback on the clarity of messages by commenting on whether the content is written in an accessible manner for the target audience (e.g., jargon-free). They could also comment on whether the medium of the message is accessible to the target-audience and suggest other forms that might facilitate broader engagement (e.g., talks, videos), including engagement of specific groups (e.g., those with co-occurring learning disabilities). The benefits here are broad—the autism community are more able to access scientific messages, thus enhancing trust between scientists and the community, and there is a greater “impact” of researchers' scientific works (which may be seen, for example, in higher Altmetric scores).

## Discussion

This article discussed the importance of participatory autism research with a particular focus on the benefits of *consultation* with the autism community. Through worked examples, we have highlighted that consultation is important throughout all stages of the research process. We appreciate that researchers might not have the means to get input from the community at all stages research (for instance if they don't have the funds to pay for this service repeatedly), and therefore we recommend that academics consider at what stages of the research process input would be most useful. Whilst we have separated our examples into different segments, it is important to note that academics can get input from the community about several parts of the research process in one consultation: for instance, in the final stages of preparing an experiment, one can get recommendations about the design of the study, the wording of participant-facing documents, recruitment, data collection and suggested dissemination routes. Of course, continual involvement of the community is preferable (rather than one instance of engagement), and therefore academics must also consider other manifestations of participatory research such as *leadership* by autistic researchers, continued *partnership* with autistic individuals, and repeated *engagement* with the community (e.g., via social media). However, we believe that, for those who are new to participatory research, consultation with the community comprises a good starting point. Regardless of its specific manifestation, autism researchers should commit to involving the autism community, thus promoting a brighter future for autistic people, together.

## Author Contributions

CTK conceptualized and wrote the article.

## Conflict of Interest

The author declares that the research was conducted in the absence of any commercial or financial relationships that could be construed as a potential conflict of interest.

## Publisher's Note

All claims expressed in this article are solely those of the authors and do not necessarily represent those of their affiliated organizations, or those of the publisher, the editors and the reviewers. Any product that may be evaluated in this article, or claim that may be made by its manufacturer, is not guaranteed or endorsed by the publisher.

## Footnotes

^1^Please note that you can email Matt Bond at m.bond@bham.ac.uk if you would like to consult the Birmingham Psychology Autism Research Team Consultancy Committee about your research.

## References

[B1] Adams D. Heussler H. Gray K. M. (2018). Behavioural phenotypes: working towards translational research through research partnerships. J. Intellect. Disabil. Res. 62, 661–663. 10.1111/jir.12513 30003626

[B2] Arnstein S. R. (1969). A ladder of citizen participation. J. Am. Inst. Plann. 35, 216–224. 10.1080/01944366908977225

[B3] Autistica (2016) Top 10 Research Priorities. London: Autistica. Available online at: https://www.autistica.org.uk/our-research/our-research/your-research-priorities (accessed May 20, 2021).

[B4] Bagatell N. (2010). From cure to community: transforming notions of autism. Ethos 38, 33–55. 10.1111/j.1548-1352.2009.01080.x

[B5] Bertilsdotter Rosqvist H. (2019). Knowing what to do: exploring meanings of development and peer support aimed at people with autism. Int. J. Inclus. Educ. 23, 174–187. 10.1080/13603116.2018.1427807

[B6] Carrington S. J. Uljarevic M. Roberts A. (2016). Knowledge acquisition and research evidence in autism: researcher and practitioner perspectives and engagement. Res. Dev. Disabil. 51, 126–134. 10.1016/j.ridd.2016.01.011 26826464

[B7] Cassidy S. A. Bradley L. Cogger-Ward H. Rodgers J. (2021b). Development and validation of the suicidal behaviours questionnaire-autism spectrum conditions in a community sample of autistic, possibly autistic and non-autistic adults. Mol. Autism 12, 1–22. 10.1186/s13229-021-00449-3 34154642 PMC8218414

[B8] Cassidy S. A. Bradley L. Cogger-Ward H. Shaw R. Bowen E. Glod M.. (2020b). Measurement properties of the suicidal behaviour questionnaire-revised in autistic adults. J. Autism Dev. Disord. 50, 3477–3488. 10.1007/s10803-020-04431-5 32125569 PMC7502048

[B9] Cassidy S. A. Goodwin J. Robertson A. Rodgers R. (2021a). INSAR Policy Brief: Autism Community Priorities for Suicide Prevention. Available online at: https://cdn.ymaws.com/www.autism-insar.org/resource/resmgr/files/policybriefs/2021-insar_policy_brief.pdf (accessed August 9, 2021).

[B10] Cassidy S. A. Robertson A. Townsend E. O'Connor R. C. Rodgers J. (2020a). Advancing our understanding of self-harm, suicidal thoughts and behaviours in autism. J. Autism Dev. Disord. 50, 3445–3449. 10.1007/s10803-020-04643-9 32880789

[B11] Chown N. Robinson J. Beardon L. Downing J. Hughes L. Leatherland J.. (2017). Improving research about us, with us: a draft framework for inclusive autism research. Disabil. Soc. 32, 720–734. 10.1080/09687599.2017.1320273

[B12] Cornwall A. Jewkes R. (1995). What is participatory research? Soc. Sci. Med. 41, 1667–1676. 10.1016/0277-9536(95)00127-S 8746866

[B13] Crane L. Adams F. Harper G. Welch J. Pellicano E. (2019). ‘Something needs to change': mental health experiences of young autistic adults in England. Autism 23, 477–493. 10.1177/1362361318757048 29415558

[B14] Dawson M. (2004). The Misbehaviour of Behaviourists. Available online at: https://www.sentex.ca/~nexus23/naa_aba.html (accessed August 9, 2021).

[B15] Fletcher-Watson S. Adams J. Brook K. Charman T. Crane L. Cusack J.. (2019). Making the future together: shaping autism research through meaningful participation. Autism 23, 943–953. 10.1177/1362361318786721 30095277 PMC6512245

[B16] Gowen E. Taylor R. Bleazard T. Greenstein A. Baimbridge P. Poole D. (2019). Guidelines for conducting research studies with the autism community. Autism Policy Pract. 2, 29–45. 32226635 PMC7099931

[B17] Grinker R. R. Chambers N. Njongwe N. Lagman A. E. Guthrie W. Stronach S.. (2012). “Communities” in community engagement: lessons learned from autism research in South Korea and South Africa. Autism Res. 5, 201–210. 10.1002/aur.1229 22566396 PMC3552431

[B18] Hemingway A. Norton L. Aarts C. (2015). Principles of lifeworld led public health practice in the UK and Sweden: reducing health inequalities. Nurs. Res. Pract. 2015:124591. 10.1155/2015/124591 25642346 PMC4302377

[B19] Lebenhagen C. (2020). Including speaking and nonspeaking autistic voice in research. Autism Adulth. 2, 128–131. 10.1089/aut.2019.0002 PMC8992839 36601567

[B20] Long J. Clarkson A. (2017). “Towards meaningful participation in research and support practice: effecting change in autism services,” in Autism and Intellectual Disability in Adults, Vol. 2, eds N. Milton and D. Martin (Hove: Pavilion Publishing), 41–45.

[B21] Long J. Panese J. Ferguson J. Hamill M. A. Miller J. (2017). Enabling voice and participation in autism services: using practitioner research to develop inclusive practice. Good Autism Pract. 18, 6–14. Available online at: https://www.researchgate.net/profile/Joseph-Long/publication/325402843_Enabling_voice_and_participation_in_autism_services_using_practitioner_research_to_develop_inclusive_practice/links/5b0c15a8aca2725783eb2c38/Enabling-voice-and-participation-in-autism-services-using-practitioner-research-to-develop-inclusive-practice.pdf (accessed August 9, 2021).

[B22] Michael C. (2021). Is being othered a co-occurring condition of autism?. Autism Adulth. 3, 118–119. 10.1089/aut.2021.0019 PMC8992897 36601468

[B23] Milton D. E. (2014). Autistic expertise: a critical reflection on the production of knowledge in autism studies. Autism 18, 794–802. 10.1177/1362361314525281 24637428

[B24] Milton D. E. Bracher M. (2013). Autistics speak but are they heard. Med. Sociol. Online 7, 61–69. Available online at: https://citeseerx.ist.psu.edu/viewdoc/download?doi=10.1.1.1083.6803&rep=rep1&type=pdf (accessed August 9, 2021).

[B25] Nelson N. Wright S. (1995). Power and Participatory Development: Theory and Practice. ITDG Publishing.

[B26] Nicolaidis C. Raymaker D. McDonald K. Dern S. Ashkenazy E. Boisclair C.. (2011). Collaboration strategies in nontraditional community-based participatory research partnerships: lessons from an academic–community partnership with autistic self-advocates. Prog. Commun. Health Partnersh. Res. Educ. Action 5, 143–150. 10.1353/cpr.2011.0022 21623016 PMC3319698

[B27] Nicolaidis C. Raymaker D. McDonald K. Dern S. Boisclair W. C. Ashkenazy E.. (2013). Comparison of healthcare experiences in autistic and non-autistic adults: a cross-sectional online survey facilitated by an academic-community partnership. J. Gen. Intern. Med. 28, 761–769. 10.1007/s11606-012-2262-7 23179969 PMC3663938

[B28] Parsons S. Cobb S. (2013). Who Chooses What I Need? Child Voice and User-Involvement in the Development of Learning Technologies for Children With Autism. EPSRC Observatory for Responsible Innovation in ICT. Available online at: http://eprints.soton.ac.uk/id/eprint/356044 (accessed August 9, 2021).

[B29] Pavlopoulou G. (2020). A good night's sleep: learning about sleep from autistic adolescents' personal accounts. Front. Psychol. 11:3597. 10.3389/fpsyg.2020.583868 33469436 PMC7814098

[B30] Pellicano E. Crane L. Gaudion K. (2017). Participatory Autism Research: A Starterpack. London: UCL Institute of Education.

[B31] Pellicano E. Dinsmore A. Charman T. (2014). What should autism research focus upon? Community views and priorities from the United Kingdom. Autism 18, 756–770. 10.1177/1362361314529627 24789871 PMC4230972

[B32] Pellicano E. Lawson W. Hall G. Mahony J. Lilley R. Davis C.. (2020). Documenting the untold histories of late-diagnosed autistic adults: a qualitative study protocol using oral history methodology. BMJ Open 10:e037968. 10.1136/bmjopen-2020-037968 32474432 PMC7264831

[B33] Pellicano E. Stears M. (2011). Bridging autism, science and society: moving toward an ethically informed approach to autism research. Autism Res. 4, 271–282. 10.1002/aur.201 21567986

[B34] Pellicano L. Dinsmore A. Charman T. (2013). A Future Made Together: Shaping Autism Research in the UK. Available online at: https://discovery.ucl.ac.uk/id/eprint/10017703 (accessed August 9, 2021).

[B35] Pickard H. Pellicano E. den Houting J. Crane L. (2021). Participatory autism research: early career and established researchers' views and experiences. Autism 23, 477–493. 10.1177/13623613211019594 34088215 PMC8750139

[B36] Redman S. Greenhalgh T. Adedokun L. Staniszewska S. Denegri S. Co-production of Knowledge Collection Steering Committee (2021). Co-production of knowledge: the future. BMJ 372:n434. 10.1136/bmj.n434 33593753 PMC7884902

[B37] Scott-Barrett J. Cebula K. Florian L. (2019). Listening to young people with autism: learning from researcher experiences. Int. J. Res. Method Educ. 42, 163–184. 10.1080/1743727X.2018.1462791

[B38] Vincent J. (2019). It's the fear of the unknown: transition from higher education for young autistic adults. Autism 23, 1575–1585. 10.1177/1362361318822498 30632780

[B39] Woods R. Waltz M. (2019). The strength of autistic expertise and its implications for autism knowledge production: a response to Damian Milton. Autonomy 1. Available online at: http://www.larryarnold.net/Autonomy/index.php/autonomy/article/view/CO2/html (accessed August 9, 2021).

[B40] Young A. Nicholas D. B. Chamberlain S. P. Suapa N. Gale N. Bailey A. J. (2019). Exploring and building autism service capacity in rural and remote regions: participatory action research in rural Alberta and British Columbia, Canada. Autism 23, 1143–1151. 10.1177/1362361318801340 30288988

